# GERBase: an online tool for high-resolution HLA allele and haplotype frequencies and linkage disequilibrium analysis in a Brazilian population

**DOI:** 10.3389/fgene.2026.1973772

**Published:** 2026-10-14

**Authors:** Jose Samuel da Silva, Gustavo Vinicius Alves, Joao Henrique Coelho Campos, Gisele Fabianne Rampim, Alberto Cardoso Martins Lima, Maria Gerbase-DeLima, Renato de Marco

**Affiliations:** 1 Instituto de Imunogenética, Associação Fundo de Incentivo à Pesquisa (IGEN/AFIP), São Paulo, Brazil; 2 Nephrology Division, Universidade Federal de São Paulo, São Paulo, Brazil

**Keywords:** allele frequencies, family-based haplotypes, histocompatibility, HLA haplotype frequencies, linkage disequilibrium

## Abstract

GERBase is a web tool that provides family-phased, high-resolution Human Leukocyte Antigen (HLA) allele and haplotype frequencies together with linkage disequilibrium (LD) metrics. The tool supports haplotype blocks defined by the user’s query, as well as stratification by self-reported race or visualization of the total admixed population. For analysis, HLA alleles were grouped by antigen recognition domain (ARD), while the web tool retained the ability to discriminate between alleles within the same group. The database contains 17,260 haplotypes from unrelated candidates for hematopoietic cell or kidney transplantation, typed at IGEN (São Paulo, Brazil), phased by segregation analysis in family studies rather than statistically inferred; 9,026 haplotypes (52.3%) are phased across all nine classical loci, and the remainder across at least two. Allele frequencies in GERBase closely track those reported for the Brazilian donor registry in the Common, Intermediate, and Well-Documented (CIWD)-Brazil catalog (r = 0.9988; P < 0.0001 across 238 alleles). This tool can be used in the daily practice of HLA laboratories, including the investigation of allele dropout, the optimization of virtual crossmatching when donor HLA typing is incomplete, and the enhancement of donor prioritization for allogeneic hematopoietic cell transplantation. GERBase is available at https://www.igen.org.br/gerbase/, does not require registration and is free to use.

## 1 Introduction

Human Leukocyte Antigen (HLA) genes are well known for their extensive allele diversity and their central role in the immune system and transplant immunology. The classical HLA loci are tightly linked, and allele and haplotype frequencies have been described for many populations (Maiers et al., 2007; Bardi et al., 2012; Gragert et al., 2013; Fabreti‐Oliveira et al., 2014; Salvadori et al., 2014; Torres et al., 2017; Costantino et al., 2017; Reis et al., 2018; Vianna et al., 2020; Gragert et al., 2026). Most of these studies do not directly observe haplotypes; they infer them statistically from unphased genotypes (Excoff et al., 1995). While this approach is efficient for high-frequency haplotypes, it has significant limitations due to HLA haplotype diversity and sample size effects, in which inference error rates are higher due to their low representation in sample datasets (Buhler et al., 2019). Currently, we are not aware of an openly queryable source of high-resolution, family-phased HLA haplotypes for Brazil.

An important characteristic of the HLA complex is the extensive linkage disequilibrium observed among many of its genes. Linkage Disequilibrium (LD) refers to the non-random association of alleles at two or more loci (Lewontin, 1964). In positive LD, alleles are inherited together more frequently than would be expected based on their individual frequencies in a population. Knowledge of HLA haplotype frequencies and LD patterns within a population is relevant not only to anthropological studies but also to transplantation, investigations of HLA associations with diseases (Trowsdale and Knight, 2013; Gragert et al., 2014), and the interpretation of HLA typing results.

Herein we describe GERBase, a free web tool built on family-phased, high-resolution (Nunes et al., 2011) HLA haplotypes from transplant candidates typed in our laboratory.

This report has two objectives. The first is to assess whether HLA allele frequencies in GERBase are concordant with those of the largest available high-resolution Brazilian HLA reference dataset, reported in the Common, Intermediate, and Well-Documented (CIWD)-Brazil catalog (Da Silva et al., 2025) from REDOME (Brazilian Donors Registry). The second is to present the online tool, highlighting its practical features and describing how this database and web tool may contribute to the routine activities of histocompatibility laboratories.

## 2 Materials and methods

### 2.1 Sample determination

The selected sample comes from the database of segregated haplotypes at the IGEN-AFIP Laboratory in São Paulo, Brazil, from 2017 to June 2026, including all candidates with two segregating haplotypes with at least two loci at high resolution; no additional exclusion criteria were applied in this study. Haplotypes were segregated based on direct observation within the immediate family in 98.5% of cases and within the extended family in 1.5% of cases. Any loci for which segregation could not be definitively determined were considered “non-segregating” loci and were not included in the haplotype database (which explains the presence of haplotypes with partial segregation). The study was reviewed and approved by the Research Ethics Committee of Hospital Maternidade Leonor Mendes De Barros, Approval No 8.458.811. The ethics committee waived the requirement for written informed consent due to the retrospective nature of the study and the use of de-identified patient data. This database contains 8,630 transplant candidates with haplotypes phased by family analysis. Fully phased haplotypes (9 loci) were observed in 4,513 individuals at high-resolution (HLA-A ∼ C ∼ B ∼ DRB3/4/5∼DRB1∼DQA1∼DQB1∼DPA1∼DPB1). Partial haplotypes were observed in 4,117 individuals with at least two loci phased at high resolution. Throughout this report, HLA-DRB3, -DRB4, and -DRB5 are treated as a single locus (DRB345); the figure of 11 loci used below refers to the genotyping kits, which type these three genes separately. Most candidates were awaiting hematopoietic cell transplantation (70.7%); the remainder, kidney transplantation (29.3%). Most were residents of southeastern Brazil (76.73%), mainly from the state of São Paulo (72.04%), followed by the northern region (10.38%); the other regions each account for less than 5%. Race in this sample was self-reported, consistent with the approach used in the Brazilian Demographic Census conducted by the Brazilian Institute of Geography and Statistics (IBGE). These patients self-identified as: 71.67% White (European descent); 15.05% Mixed (admixed); 3.97% Black (Sub-Saharan African descent); 0.70% Yellow (Asian descent); 0.05% Indigenous (Native American descent); and 8.56% did not declare race.

### 2.2 HLA typing

The HLA typing methodologies used in the sample varied over the years. For all samples included in the subset with 9 available loci, the typing was performed using Next-Generation Sequencing methodology. Most results were generated using the kits AllType 11 Loci (One Lambda - Thermo Fisher Scientific), followed by AlloSeq Tx 17 (CareDx-Werfen), Holotype HLA (Omixon-Werfen) and Mia Fora (Immucor-Werfen), all sequenced on the MiSeq Platform (Illumina). Over the last 2 years, the DNBSEQ-G99 (MGI Tech) and PromethION 2 Solo (Oxford Nanopore Technologies) platforms have also been used with AllType 11 Loci (One Lambda-Thermo Fisher Scientific). Among the samples with partial haplotypes, in some cases it was not possible to determine the haplotype through family analysis, while in the remaining cases, HLA typing for the 11 loci was unavailable. In these cases, typing was performed by sequence-based typing (SBT) with the SeCore kit (Thermo Fisher Scientific). All methods and kits underwent internal validation and external proficiency testing, and no relevant HLA typing discrepancies were observed at the resolution used for analysis.

### 2.3 Normalization of HLA results to ARD (g) groups

Ambiguous results reduced to the third or second field, P groups, and G groups were normalized to two-field ARD (antigen recognition domain) allele groups, as implemented in py-ard (Maiers et al., 2023) with IPD-IMGT/HLA release 3.63.0 (Robinson et al., 2024). All results are grouped into ARD groups containing alleles with variations outside the antigen recognition region. In this representation, a lowercase “g”is added after the second field to the “owner” of the group (e.g., A*02:01 g).

### 2.4 Population genetics and comparative analyses

#### 2.4.1 Determination of allelic or haplotypic frequencies and LD metrics

Frequencies were obtained by direct counting. For each query, the denominator was the number of haplotypes informative for all loci included in that query—that is, 2n for the subset of patients with phased haplotypes across those specific loci—so that denominators vary between queries and are displayed alongside each result. The LD metric used was the Relative Disequilibrium (D′) proposed by Lewontin (Lewontin, 1964), which normalizes the difference between observed frequencies and the expected frequencies (random association between alleles), such that D′ can take values between −1 and 1. Association between allele pairs was tested by Fisher’s exact test whenever any expected cell count was below 5, and by the chi-square test otherwise; the resulting P value is displayed alongside D'. P values adjusted by the Holm–Bonferroni method (Holm, 1979) are also presented. Calculations were performed using the total sample and by self-reported group (White, Mixed, and Black). The remaining groups were included only in the total sample due to their sample size. Haplotype diversity (expected heterozygosity) (Nei, 1973) was calculated for haplotypes at the 9 loci, as well as for the combination A ∼ B ∼ DRB1.

#### 2.4.2 Comparison of HLA allele frequencies between GERBase and sample representative of the Brazilian population

The allelic frequencies observed at GERBase were compared with those observed among voluntary bone marrow donors in the Brazilian registry (REDOME), as reported in the CIWD-Brazil publication (Da Silva et al., 2025), both grouped at the ARD (“g”)level. Comparisons were performed for all alleles with a frequency greater than 0.1% at GERBase (238 alleles), using contingency tables and the chi-square test with Holm–Bonferroni correction per locus. The HLA-DRB3, -DRB4, and -DRB5 genes are not mentioned in the CIWD-Brazil publication and were therefore not included in this comparison. Results below 0.1% were grouped as “other” for each locus; “other” accounted for 2.2% at HLA-B, 1.1% at HLA-A, and less than 1% at all remaining loci. Pearson’s correlation was also used to examine the overall pattern between the two databases. A comparison to identify common alleles (frequencies >1/10,000) present in CIWD-Brazil but absent from GERBase was conducted to characterize discrepancies between common alleles in the Brazilian population that are not included in this haplotype database.

### 2.5 Architecture and concept development of the online interactive query tool

GERBase was developed in Python (v3.12) using a modular architecture that separates data processing, domain logic, and API services. Data preparation was implemented as a reproducible pipeline using Polars, encompassing data cleaning, normalization, validation, and the generation of versioned datasets in Parquet format. Query and analysis functionalities were made available through a RESTful API built with FastAPI. To support data integrity, reproducibility, and traceability, each data release includes metadata, schema validation, record counts, and SHA-256 checksums. For security reasons, access may be blocked based on the user’s geolocation.

All queries evaluate only the loci for which specific data are available, excluding missing results from calculations. Thus, the database utilizes both partially and fully phased haplotypes. The online tool also includes the ability to view the alleles found in the database grouped into a specific ARD (g) group. This functionality allows for the identification of specific haplotypes containing alleles with second-field differences outside the antigen recognition region or even third-field variations in HLA nomenclature. In addition to observed haplotypic frequencies (HFO), the tool also presents expected haplotypic frequencies (HFE) under linkage equilibrium. D′ values and the statistical significance of each allele-pair association are also reported, together with a schematic color scale that identifies the pairs in significant LD. Haplotypes can also be stratified according to self-reported race.

Since the tool interacts dynamically with specific haplotype combinations, haplotypes need not be defined at all nine loci. This flexibility substantially increases the database size, as many cases do not include typing for all 9 loci or cannot be defined in family analysis for all loci. With this partial approach, the haplotype database contains 8,630 patients with haplotypes segregated across at least two loci in high resolution, totaling 17,260 haplotypes. The results presented in the tool are always guided by the loci and/or alleles searched for in the user’s query. Built with the intention of searching for haplotypes and LD metrics, the tool presents data based on search interaction. The search works for alleles and loci at different query resolutions. The character “?” is used to include a locus without specifying an allele, in which case all possibilities will be presented.

For the HLA-DRB3, -DRB4, and -DRB5 genes, queries can be made individually or using the special query “DRB345*”including the absence of them, which is represented as “DRBx-Absent”. For all calculations, the alleles of these three genes were considered to belong to a single locus (DRB345), with the absence being one of the “allele” possibilities for this locus.

## 3 Results

### 3.1 Haplotype composition

Of the 17,260 haplotypes in the database, 9,026 (52.29%) are phased across all nine loci, and 67.39% are phased across HLA-A, -C, -B, -DRB1, and -DQB1. Because queries evaluate only the loci for which data are available, the denominator differs between queries and is displayed with every result. Among the 9,026 fully phased haplotypes, 4,562 were observed only once (haplotype diversity 0.99972); for A ∼ B ∼ DRB1, 2,768 of 13,340 informative haplotypes were observed once (haplotype diversity 0.99879).

### 3.2 Comparison of HLA allele frequencies between GERBase and REDOME

The allele frequencies (ARD level) calculated for all loci and compared with the frequencies reported in the CIWD-Brazil publication are available in Supplementary Material 1. Seventeen of the 238 alleles compared across eight loci differed significantly between the two datasets after Holm–Bonferroni correction (Table 1). Across all compared alleles, Pearson’s correlation was 0.9988 and the slope of the linear regression was close to unity (y = 1.0049x - 0.0159).

**TABLE 1:** HLA alleles with significant differences in frequencies between GERBase and CIWD-Brazil.

GERBase alleles (ARD)	N GERBase (% freq.)	N CIWD-Brazil (% freq.)	P Value (holm–Bonferroni)
A*02:11 g	172 (1.23)	5,097 (0.85)	<0.0001
B*35:05 g	130 (0.91)	3,752 (0.63)	0.0025
B*46:01 g	15 (0.11)	160 (0.03)	<0.0001
B*48:01 g	22 (0.15)	362 (0.06)	0.0017
B*50:01 g	235 (1.65)	12,333 (2.07)	0.0386
B*54:01 g	21 (0.15)	210 (0.04)	<0.0001
C*01:02 g	360 (2.77)	13,537 (2.32)	0.023
C*08:03 g	26 (0.2)	526 (0.09)	0.0023
DQA1*01:03 g	868 (6.74)	14,249 (7.52)	0.0097
DQA1*02:01 g	1,445 (11.22)	23,782 (12.55)	<0.0001
DQA1*03:01 g	2098 (16.29)	28,147 (14.85)	<0.0001
DQA1*04:01 g	963 (7.48)	12,220 (6.45)	<0.0001
DQB1*02:01 g	3,041 (19.15)	119,800 (20.53)	0.0003
DQB1*04:01 g	32 (0.2)	395 (0.07)	<0.0001
DQB1*06:03 g	915 (5.76)	37,939 (6.5)	0.0028
DRB1*07:01 g	1890 (11.26)	75,712 (12.7)	<0.0001
DRB1*08:04 g	373 (2.22)	11,158 (1.87)	0.0441
DRB1*13:01 g	1,061 (6.32)	41,632 (6.98)	0.0426

Among the alleles common in the CIWD-Brazil catalog but absent from GERBase, only 5 alleles were found, all with frequencies between 0.01% and 0.02%. They are HLA-B*08:18, HLA-C*07:35, HLA-DQB1*03:106, HLA-DRB1*13:14, and HLA-DRB1*14:17.

### 3.3 Haplotype frequencies

Table 2 presents the top 20 haplotypes for the HLA-A, -B, -C, -DRB1, and -DQB1 as examples of the most common ones; however, the intention is for the reader to be able to explore the haplotypes and linkage disequilibrium metrics for any haplotype present in the database and for any combination of loci using the online tool, which will be described in more detail in the next section of this article.

**TABLE 2:** Top 20 HLA haplotypes at GERBase.

A ∼ C ∼ B ∼ DRB1∼DQB1 haplotypes	N GERBase (% freq.)
A*01:01 g ∼ C*07:01 g ∼ B*08:01 g ∼ DRB1*03:01 g ∼ DQB1*02:01 g	184 (1.58)
A*29:02 g ∼ C*16:01 g ∼ B*44:03 g ∼ DRB1*07:01 g ∼ DQB1*02:01 g	128 (1.1)
A*33:01 g ∼ C*08:02 g ∼ B*14:02 g ∼ DRB1*01:02 g ∼ DQB1*05:01 g	95 (0.82)
A*03:01 g ∼ C*07:02 g ∼ B*07:02 g ∼ DRB1*15:01 g ∼ DQB1*06:02 g	85 (0.73)
A*11:01 g ∼ C*04:01 g ∼ B*35:01 g ∼ DRB1*01:01 g ∼ DQB1*05:01 g	57 (0.49)
A*02:01 g ∼ C*07:02 g ∼ B*07:02 g ∼ DRB1*15:01 g ∼ DQB1*06:02 g	53 (0.46)
A*03:01 g ∼ C*04:01 g ∼ B*35:01 g ∼ DRB1*01:01 g ∼ DQB1*05:01 g	48 (0.41)
A*02:01 g ∼ C*05:01 g ∼ B*44:02 g ∼ DRB1*13:01 g ∼ DQB1*06:03 g	46 (0.4)
A*02:11 g ∼ C*04:01 g ∼ B*48:02 g ∼ DRB1*09:01 g ∼ DQB1*03:03 g	43 (0.37)
A*23:01 g ∼ C*04:01 g ∼ B*44:03 g ∼ DRB1*07:01 g ∼ DQB1*02:01 g	43 (0.37)
A*02:01 g ∼ C*14:02 g ∼ B*51:01 g ∼ DRB1*08:01 g ∼ DQB1*04:02 g	42 (0.36)
A*68:01 g ∼ C*03:04 g ∼ B*40:04 g ∼ DRB1*04:11 g ∼ DQB1*03:02 g	38 (0.33)
A*30:01 g ∼ C*06:02 g ∼ B*13:02 g ∼ DRB1*07:01 g ∼ DQB1*02:01 g	37 (0.32)
A*30:02 g ∼ C*05:01 g ∼ B*18:01 g ∼ DRB1*03:01 g ∼ DQB1*02:01 g	36 (0.31)
A*24:02 g ∼ C*04:01 g ∼ B*35:02 g ∼ DRB1*11:04 g ∼ DQB1*03:01 g	35 (0.3)
A*24:02 g ∼ C*07:02 g ∼ B*07:02 g ∼ DRB1*15:01 g ∼ DQB1*06:02 g	35 (0.3)
A*02:01 g ∼ C*08:02 g ∼ B*14:02 g ∼ DRB1*01:02 g ∼ DQB1*05:01 g	34 (0.29)
A*02:01 g ∼ C*05:01 g ∼ B*18:01 g ∼ DRB1*03:01 g ∼ DQB1*02:01 g	34 (0.29)
A*02:01 g ∼ C*05:01 g ∼ B*44:02 g ∼ DRB1*04:01 g ∼ DQB1*03:01 g	34 (0.29)
A*02:01 g ∼ C*03:03 g ∼ B*15:04 g ∼ DRB1*16:02 g ∼ DQB1*03:01 g	34 (0.29)

The sample size for these 5 loci is 11,631 haplotypes.

### 3.4 GERBase–online interactive tool for HLA haplotypes and LD metrics

To display allele or haplotype frequencies, as well as LD metrics, the tool requires the user to specify loci or alleles; it then returns the data for that specific allele and/or locus queried. If the special character “?” is used, all results will be displayed; for example, in the search “C*?”, the allelic frequencies of HLA-C will be shown. Figure 1 uses this example to illustrate HLA-C frequencies, as well as to show stratification by race and ARD cluster. To view haplotypes and LD metrics, the user need only enter two loci. In the previous example of “C*?”, if the allele “B*51:01”is included in the query, the tool then shows all haplotypes containing HLA-B*51:01 with any HLA-C allele. This example is shown in Figure 2 to illustrate the metrics and format used to present the results in the tool. Allele searches performed by users in the tool are always directed toward ARD group searches; therefore, if a user searches for an allele within a group, the result will always be displayed for the ARD group searched, even if the allele itself does not exist in the database. A specific search for an allele within the ARD group present in the database can be performed after the initial query, in a second search field that becomes available.

**FIGURE 1:**
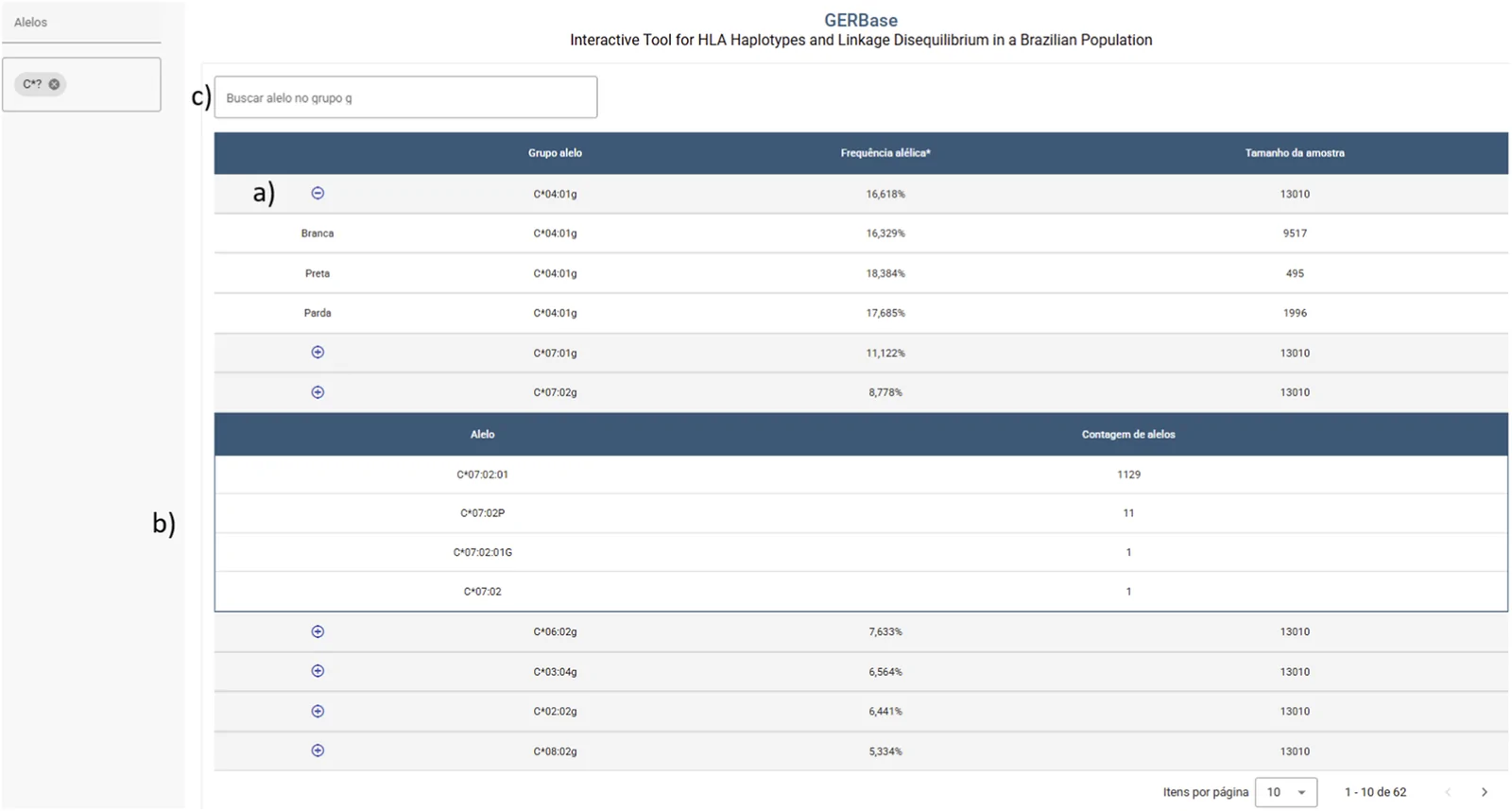
Example in the online tool of HLA-C allelic frequencies. Notes: In the queries the special character “?” is used to list all alleles of one locus. Stratification by self-reported race is obtained by clicking the “+” at the beginning of each row. **(a)** To view the alleles in the database that were grouped by ARD (“g”), the user simply clicks on a haplotype row, and all combinations present in that haplotype will be displayed. **(b)** To search for alleles within the listed ARD groups, use the internal search field **(c)**.

**FIGURE 2:**
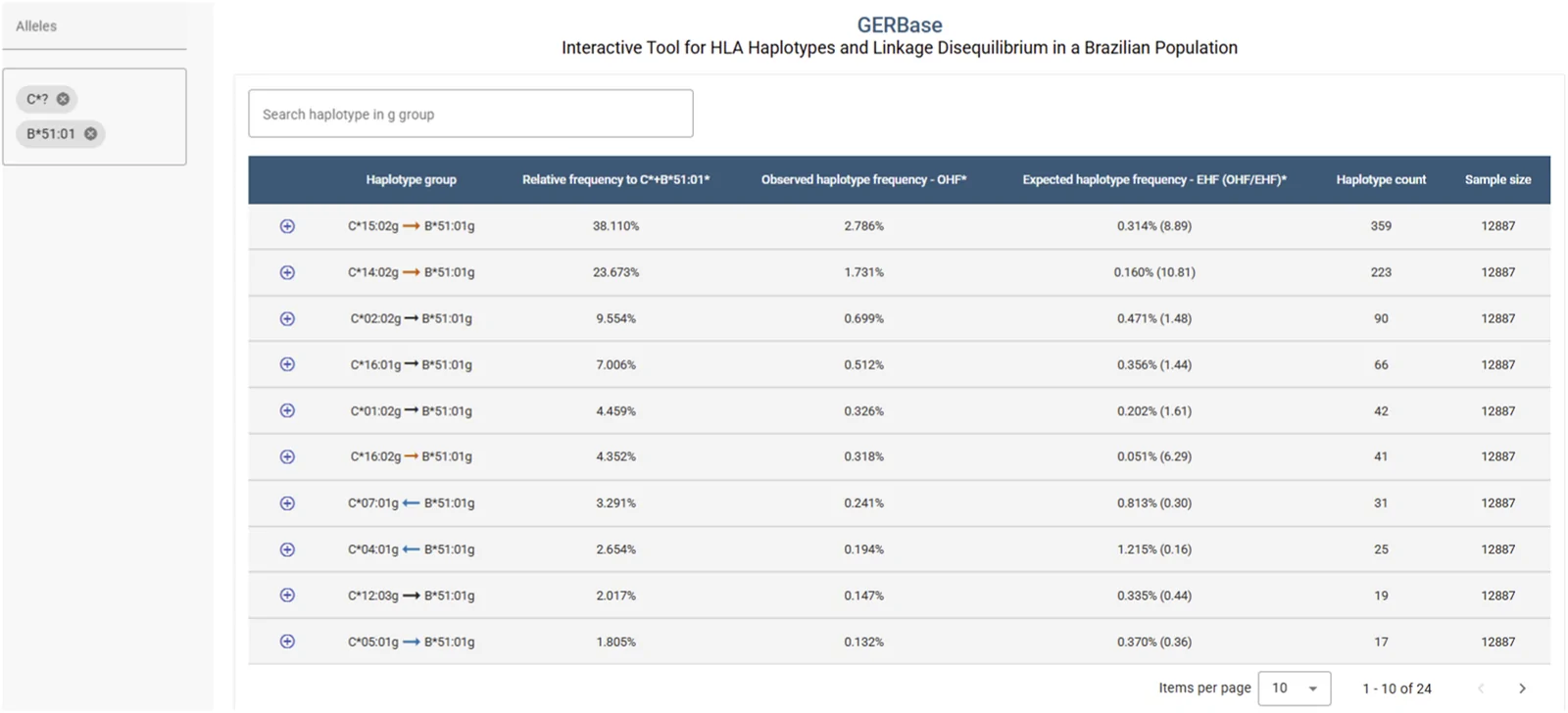
Example of query in the online tool for C ∼ B haplotypes with HLA-B*51:01. Notes: When more than one locus or alleles from different loci are included, the tool returns the haplotypes from the query. The relative frequency column shows the frequency within the haplotypes present in the query. The observed frequency represents the haplotype frequencies in the database. The column expected frequency is the haplotype frequency calculated under linkage equilibrium (random association between alleles). Sample size shows the number of informative haplotypes for that query. The color-coded arrows is used to simply distinguish haplotypes that are in equilibrium (black arrows, p > 0.05) from those in linkage disequilibrium (p < 0.05); orange for D′ positive and blue for D′ negative. The direction of the arrow always points toward the allele with higher frequency.

Two of these outputs need explanation. The relative frequency is the frequency within the set of haplotypes returned by the query, whereas the observed frequency (HFO) refers to the database. In the query shown in Figure 2, among C ∼ B haplotypes carrying B*51:01 with any HLA-C allele, C*15:02 g ∼ B*51:01 g accounts for 38.11% of the returned haplotypes but only 2.79% of the database.

The HFE is displayed because it is the quantity against which the HFO is compared: it is the frequency the haplotype would have under linkage equilibrium (Lewontin, 1964; Lewontin, 1988), and the departure between the two is what the arrows in the haplotype view represent. The remaining elements of the output are summarized in Table 3.

**TABLE 3:** Features presented at web tool.

Interface element	What it reports and how it is computed
Observed frequency	Frequency of the haplotype in the database, by direct counting over all haplotypes informative for the queried loci
Relative frequency	Frequency of the haplotype within the set returned by the query; usable as an empirical probability for a locus left open with “?”
Expected haplotype frequency	Frequency expected under linkage equilibrium, from the product of the individual allele frequencies; the ratio of observed to expected frequency is shown alongside it
Sample size	Number of haplotypes informative for every locus in that query; it is the denominator of all frequencies in the row and varies between queries
Arrow color	Black, allele pair in linkage equilibrium (P > 0.05); orange, positive D′; blue, negative D′ (P < 0.05). Colors follow the Holm–Bonferroni-adjusted P value, which is computed over the possible pairs returned by the query of the two loci
Arrow direction	Points from the less to the more frequent allele of the pair, so that the asymmetry between the two allele frequencies is visible without reading the numbers
Arrow length	Proportional to |D′|; pairs at |D′| = 1 are drawn longest
Arrow hover window	D′, the unadjusted P value (chi-square or Fisher′s exact test) and the Holm–Bonferroni-adjusted P value for that allele pair
Race stratification	Expands the row into the self-reported white, mixed, and black groups, each with its own denominator
ARD group expansion	Lists the alleles observed within the queried ARD (″g″) group, including second- and third-field variants

## 4 Discussion

GERBase was built around one design decision: the user defines which HLA loci are displayed, not the tool. A laboratory interested in any two-locus block need not read through an entire nine-locus haplotype, and a query combining a specific allele with the wildcard “?” at another locus returns the distribution of possibilities at the open locus. In this type of analysis, the relative frequency can be interpreted as the empirical probability of a given combination, which is useful for predicting a locus that was not typed and for narrowing high-resolution possibilities from older low-resolution results.

GERBase differs from the static haplotype tables of individual publications, and from established resources such as the Allele Frequency Net Database (Santos et al., 2016) and HaploStats (HaploStats, 2026), in three respects: haplotypes are family-phased rather than estimated by Expectation-Maximization (EM); LD metrics are provided; and the alleles contained within each ARD group remain inspectable alongside the group frequency.

One concern in building the tool on this haplotype database was whether transplant candidates adequately represent the Brazilian population. Compared to the largest available Brazilian high-resolution reference dataset (Da Silva et al., 2025), allele frequencies in GERBase closely track those of the REDOME registry (r = 0.9988, P < 0.0001 across 238 alleles), with statistically significant but small differences at 17 alleles (Table 1). Five alleles classified as common in CIWD-Brazil were absent; all these alleles fall at 0.01%–0.02%, so their absence is consistent with sampling rather than indicating a coverage gap. Regional variation already documented for HLA haplotypes in Brazil (Fabreti‐Oliveira et al., 2014; Torres et al., 2017) may be present, but was not evaluated here.

Most published Brazilian datasets report low-resolution haplotypes (Bardi et al., 2012; Salvadori et al., 2014; Torres et al., 2017; Costantino et al., 2017; Reis et al., 2018) or imputed high-resolution haplotypes (Halagan et al., 2018), and the high-resolution studies rest on smaller samples with imputed phase (Vianna et al., 2020). For common haplotypes, and in registries of millions of individuals, EM estimation is accurate. However, with the HLA diversity in small samples, the concordance can be very low. Buhler et al. (Buhler et al., 2019) reported that only 39% of haplotypes matched between family-segregated assignments and those imputed by the EM method for HLA-A ∼ B ∼ DRB1. The contribution of family phasing in a dataset where many haplotypes were observed only once is that each haplotype was defined by allele segregation and cannot be the result of inference errors.

One application of the GERBase tool could be to search for common haplotypes and investigate possible dropouts. In one case observed in our Laboratory, a multiplex HLA genotyping (11 loci) returned an apparently homozygous HLA-DQA1*01:02 result together with HLA-DRB1*07:01 and HLA-DQB1*02:02. A GERBase query showed that DRB1*07:01∼DQB1*02:02 haplotypes in this population almost invariably carry HLA-DQA1*02:01, making the observed combination improbable and suggesting allele dropout; repeat testing confirmed an HLA-DQA1*02:01 allele that the first assay had failed to amplify. This practical example illustrates the value of relative frequencies as a plausibility step, although they do not provide a quantification.

Another use can be applied when performing virtual crossmatch (VXM) assessment with incomplete donor HLA typing information, ultimately informing potential negative and positive VXM results. For instance, a transplant candidate showed strong reactivity to HLA-C*06:02. The donor, who had HLA-B*35/-B*51, did not have HLA-C data available or DNA for extended typing. To address this, we entered the donor’s HLA-B information into GERBase and interrogated the query for HLA-C. The probability that a donor carrying HLA-B*35/-B*51 also carried HLA-C*06:02 was found to be very low (<1%), suggesting a negative VXM. Hence, the medical team decided to accept the organ offer. On the other hand, when a patient exhibiting strong Cw0401 antibodies was analyzed against this same donor, GERBase highlighted a very high probability (>85%) of a Cw0401 donor-specific antibody (DSA). In this case, the medical team could deny the offer due to the likely positive VXM. These examples demonstrate the usefulness of GERBase for addressing common issues in DSA analysis in the HLA laboratory.

Our tool may also be used for patients eligible for allogeneic hematopoietic cell transplantation (HCT) who undergo searches for unrelated donors and umbilical cord blood units. For instance, in the Brazilian Unrelated Donor Registry, search reports may contain only data on HLA-A, -B, and -DRB1 at medium resolution for all available unrelated donors. This limitation is also common in the umbilical cord blood setting. In these cases, GERBase can help infer missing HLA-C and HLA-DQB1 alleles in the donor/recipient pair, thereby informing which unrelated donors or cord blood units should be prioritized for high-resolution HLA typing. Furthermore, in HCT patients showing substantial allosensitization to HLA and with only donor search reports with incomplete HLA typing, GERBase may be useful for anticipating potential DSAs against HLA alleles absent from the unrelated donor and umbilical cord blood searches.

We acknowledge that the GERBase tool presents some limitations. Its database is small relative to the diversity of the HLA system and contains fewer haplotypes than studies based on large donor registries (Maiers et al., 2007; Gragert et al., 2013; Gragert et al., 2026; Halagan et al., 2018); this limits our ability to detect haplotypes carrying intermediate, well-documented, or rare alleles. Race is self-reported using IBGE categories, which may be influenced by inherent reporting biases (Pena et al., 2011). Frequencies and LD were calculated at the ARD level, meaning that grouping affects the LD estimates, not just the displayed resolution. The ability to inspect alleles within each ARD group can aid in manually addressing this issue. Intraregional variations within Brazil or comparisons with populations worldwide were not evaluated. Despite these limitations, GERBase provides a complementary, observation-based view of haplotype structure and linkage disequilibrium in a Brazilian population.

In conclusion, the GERBase tool, which is based solely on segregated haplotypes from transplant candidates, effectively captures the major haplotypes present in the Brazilian population and can serve as a complementary resource for consulting frequency data. While HLA haplotype tools exist, our tool offers not only haplotype information but also an actionable view of relative frequencies. Furthermore, its practical applications can optimize several daily practices activities within the HLA Laboratory, in both solid organ transplantation and allogeneic HCT. Lastly, this tool is accessible at https://www.igen.org.br/gerbase/, requires no registration, and is available free of charge. Annual updates of database will be available.

## Data Availability

The original contributions presented in the study are publicly available. The dataset can be accessed in Zenodo with the DOI: 10.5281/zenodo.22959989.
