# Supplementary material for: GERBase: an online tool for high-resolution HLA allele and haplotype frequencies and linkage disequilibrium analysis in a Brazilian population
Source: Front Genet. 2026 Oct 14;17:1973772. doi: 10.3389/fgene.2026.1973772 (PMC13713022; doi:10.3389/fgene.2026.1973772)
Supplement: Supplementary file 1 [file Table1.docx]

**Supplementary Material 1:** HLA alleles frequencies at GERBase compared to CIWD-Brazil.

| **GERBase Alleles (ARD)** | **N GERBase (% Freq.)** | **N CIWD-Brazil (% Freq.)** | ***p value* (Holm-Bonferroni)** |
| --- | --- | --- | --- |
| A*01:01g | 1081 (7.76) | 49813 (8.35) |  |
| A*01:02g | 39 (0.28) | 1291 (0.22) |  |
| A*01:03g | 2 (0.01) | 97 (0.02) |  |
| A*01:482g | 1 (0.01) | 0 (<0.01) |  |
| A*02:01g | 2838 (20.37) | 125439 (21.04) |  |
| A*02:02g | 141 (1.01) | 6518 (1.09) |  |
| A*02:03g | 1 (0.01) | 53 (0.01) |  |
| A*02:04g | 71 (0.51) | 2751 (0.46) |  |
| A*02:05g | 166 (1.19) | 8237 (1.38) |  |
| A*02:06g | 22 (0.16) | 526 (0.09) |  |
| A*02:07g | 6 (0.04) | 146 (0.02) |  |
| A*02:08g | 1 (0.01) | 13 (<0.01) |  |
| A*02:11g | 172 (1.23) | 5097 (0.85) | <0.0001 |
| A*02:12g | 4 (0.03) | 113 (0.02) |  |
| A*02:13g | 5 (0.04) | 38 (0.01) |  |
| A*02:14g | 1 (0.01) | 78 (0.01) |  |
| A*02:158g | 1 (0.01) | 73 (0.01) |  |
| A*02:17g | 9 (0.06) | 178 (0.03) |  |
| A*02:20g | 13 (0.09) | 607 (0.1) |  |
| A*02:22g | 13 (0.09) | 278 (0.05) |  |
| A*02:33g | 4 (0.03) | 48 (0.01) |  |
| A*02:52g | 5 (0.04) | 146 (0.02) |  |
| A*02:890g | 1 (0.01) | 9 (<0.01) |  |
| A*03:01g | 1145 (8.22) | 50229 (8.42) |  |
| A*03:02g | 52 (0.37) | 1910 (0.32) |  |
| A*03:05g | 1 (0.01) | 15 (<0.01) |  |
| A*11:01g | 712 (5.11) | 30202 (5.06) |  |
| A*11:02g | 1 (0.01) | 3 (<0.01) |  |
| A*11:08g | 1 (0.01) | 19 (<0.01) |  |
| A*11:09g | 1 (0.01) | 0 (<0.01) |  |
| A*11:12g | 2 (0.01) | 40 (0.01) |  |
| A*23:01g | 760 (5.46) | 33152 (5.56) |  |
| A*23:05g | 12 (0.09) | 518 (0.09) |  |
| A*23:15g | 1 (0.01) | 8 (<0.01) |  |
| A*24:02g | 1243 (8.92) | 50140 (8.41) |  |
| A*24:03g | 158 (1.13) | 5694 (0.95) |  |
| A*24:05g | 4 (0.03) | 162 (0.03) |  |
| A*24:06g | 1 (0.01) | 22 (<0.01) |  |
| A*24:07g | 2 (0.01) | 136 (0.02) |  |
| A*24:160g | 1 (0.01) | 0 (<0.01) |  |
| A*24:26g | 5 (0.04) | 144 (0.02) |  |
| A*24:30g | 1 (0.01) | 56 (0.01) |  |
| A*25:01g | 164 (1.18) | 6778 (1.14) |  |
| A*26:01g | 392 (2.81) | 16941 (2.84) |  |
| A*26:02g | 2 (0.01) | 98 (0.02) |  |
| A*26:03g | 4 (0.03) | 73 (0.01) |  |
| A*26:08g | 25 (0.18) | 1012 (0.17) |  |
| A*26:12g | 1 (0.01) | 49 (0.01) |  |
| A*26:27g | 1 (0.01) | 15 (<0.01) |  |
| A*26:31g | 1 (0.01) | 50 (0.01) |  |
| A*29:01g | 25 (0.18) | 1356 (0.23) |  |
| A*29:02g | 552 (3.96) | 25105 (4.21) |  |
| A*29:10g | 1 (0.01) | 15 (<0.01) |  |
| A*29:15g | 1 (0.01) | 0 (<0.01) |  |
| A*30:01g | 367 (2.63) | 16058 (2.69) |  |
| A*30:02g | 404 (2.9) | 17500 (2.93) |  |
| A*30:04g | 41 (0.29) | 1596 (0.27) |  |
| A*30:09g | 1 (0.01) | 25 (<0.01) |  |
| A*30:10g | 1 (0.01) | 43 (0.01) |  |
| A*30:153g | 1 (0.01) | 6 (<0.01) |  |
| A*31:01g | 697 (5) | 27777 (4.66) |  |
| A*31:02g | 6 (0.04) | 121 (0.02) |  |
| A*31:15g | 26 (0.19) | 1085 (0.18) |  |
| A*32:01g | 401 (2.88) | 17961 (3.01) |  |
| A*32:03g | 1 (0.01) | 2 (<0.01) |  |
| A*33:01g | 313 (2.25) | 11939 (2) |  |
| A*33:03g | 180 (1.29) | 6775 (1.14) |  |
| A*33:05g | 3 (0.02) | 143 (0.02) |  |
| A*34:01g | 1 (0.01) | 5 (<0.01) |  |
| A*34:02g | 131 (0.94) | 5702 (0.96) |  |
| A*36:01g | 92 (0.66) | 3731 (0.63) |  |
| A*43:01g | 4 (0.03) | 47 (0.01) |  |
| A*66:01g | 120 (0.86) | 5394 (0.9) |  |
| A*66:02g | 23 (0.17) | 1131 (0.19) |  |
| A*66:03g | 8 (0.06) | 324 (0.05) |  |
| A*68:01g | 569 (4.08) | 22665 (3.8) |  |
| A*68:02g | 379 (2.72) | 15635 (2.62) |  |
| A*68:05g | 1 (0.01) | 36 (0.01) |  |
| A*68:16g | 1 (0.01) | 0 (<0.01) |  |
| A*68:17g | 15 (0.11) | 314 (0.05) |  |
| A*68:23g | 1 (0.01) | 2 (<0.01) |  |
| A*69:01g | 23 (0.17) | 1015 (0.17) |  |
| A*74:01g | 210 (1.51) | 8761 (1.47) |  |
| A*74:03g | 7 (0.05) | 344 (0.06) |  |
| A*80:01g | 35 (0.25) | 1398 (0.23) |  |
| B*07:02g | 829 (5.82) | 37113 (6.22) |  |
| B*07:05g | 82 (0.58) | 3338 (0.56) |  |
| B*07:09g | 2 (0.01) | 82 (0.01) |  |
| B*07:14g | 4 (0.03) | 183 (0.03) |  |
| B*07:20g | 3 (0.02) | 134 (0.02) |  |
| B*07:310g | 1 (0.01) | 2 (<0.01) |  |
| B*07:48g | 1 (0.01) | 1 (<0.01) |  |
| B*08:01g | 613 (4.3) | 27754 (4.65) |  |
| B*08:12g | 1 (0.01) | 8 (<0.01) |  |
| B*13:01g | 7 (0.05) | 111 (0.02) |  |
| B*13:02g | 198 (1.39) | 8357 (1.4) |  |
| B*14:01g | 143 (1) | 6265 (1.05) |  |
| B*14:02g | 639 (4.48) | 24739 (4.15) |  |
| B*14:03g | 12 (0.08) | 465 (0.08) |  |
| B*15:01g | 387 (2.71) | 16563 (2.78) |  |
| B*15:02g | 1 (0.01) | 185 (0.03) |  |
| B*15:03g | 302 (2.12) | 12412 (2.08) |  |
| B*15:04g | 123 (0.86) | 4803 (0.81) |  |
| B*15:05g | 18 (0.13) | 629 (0.11) |  |
| B*15:07g | 6 (0.04) | 254 (0.04) |  |
| B*15:08g | 46 (0.32) | 1715 (0.29) |  |
| B*15:09g | 16 (0.11) | 611 (0.1) |  |
| B*15:107g | 1 (0.01) | 1 (<0.01) |  |
| B*15:10g | 188 (1.32) | 7639 (1.28) |  |
| B*15:11g | 4 (0.03) | 30 (0.01) |  |
| B*15:13g | 1 (0.01) | 23 (<0.01) |  |
| B*15:15g | 8 (0.06) | 233 (0.04) |  |
| B*15:16g | 58 (0.41) | 2652 (0.44) |  |
| B*15:17g | 112 (0.79) | 5119 (0.86) |  |
| B*15:18g | 46 (0.32) | 1881 (0.32) |  |
| B*15:20g | 69 (0.48) | 2345 (0.39) |  |
| B*15:21g | 1 (0.01) | 7 (<0.01) |  |
| B*15:23g | 1 (0.01) | 17 (<0.01) |  |
| B*15:24g | 3 (0.02) | 124 (0.02) |  |
| B*15:27g | 2 (0.01) | 13 (<0.01) |  |
| B*15:31g | 12 (0.08) | 332 (0.06) |  |
| B*15:33g | 1 (0.01) | 86 (0.01) |  |
| B*15:37g | 3 (0.02) | 180 (0.03) |  |
| B*15:38g | 1 (0.01) | 7 (<0.01) |  |
| B*15:39g | 5 (0.04) | 123 (0.02) |  |
| B*15:405g | 2 (0.01) | 2 (<0.01) |  |
| B*15:47g | 12 (0.08) | 311 (0.05) |  |
| B*15:599g | 1 (0.01) | 1 (<0.01) |  |
| B*15:614g | 1 (0.01) | 2 (<0.01) |  |
| B*15:71g | 1 (0.01) | 2 (<0.01) |  |
| B*18:01g | 643 (4.51) | 24771 (4.15) |  |
| B*18:03g | 4 (0.03) | 227 (0.04) |  |
| B*18:04g | 7 (0.05) | 191 (0.03) |  |
| B*18:05g | 7 (0.05) | 509 (0.09) |  |
| B*18:06g | 1 (0.01) | 35 (0.01) |  |
| B*18:14g | 1 (0.01) | 2 (<0.01) |  |
| B*18:19g | 1 (0.01) | 6 (<0.01) |  |
| B*18:20g | 1 (0.01) | 1 (<0.01) |  |
| B*18:25g | 1 (0.01) | 17 (<0.01) |  |
| B*27:02g | 30 (0.21) | 1230 (0.21) |  |
| B*27:03g | 12 (0.08) | 548 (0.09) |  |
| B*27:04g | 1 (0.01) | 6 (<0.01) |  |
| B*27:05g | 258 (1.81) | 9975 (1.67) |  |
| B*27:07g | 3 (0.02) | 258 (0.04) |  |
| B*27:08g | 3 (0.02) | 230 (0.04) |  |
| B*27:09g | 7 (0.05) | 58 (0.01) |  |
| B*35:01g | 728 (5.11) | 32126 (5.39) |  |
| B*35:02g | 197 (1.38) | 7747 (1.3) |  |
| B*35:03g | 245 (1.72) | 10968 (1.84) |  |
| B*35:04g | 126 (0.88) | 4068 (0.68) |  |
| B*35:05g | 130 (0.91) | 3752 (0.63) | 0.0025 |
| B*35:06g | 24 (0.17) | 663 (0.11) |  |
| B*35:08g | 137 (0.96) | 5733 (0.96) |  |
| B*35:09g | 2 (0.01) | 25 (<0.01) |  |
| B*35:10g | 7 (0.05) | 225 (0.04) |  |
| B*35:11g | 30 (0.21) | 905 (0.15) |  |
| B*35:12g | 1 (0.01) | 6 (<0.01) |  |
| B*35:19g | 1 (0.01) | 22 (<0.01) |  |
| B*35:20g | 31 (0.22) | 1342 (0.23) |  |
| B*35:21g | 1 (0.01) | 68 (0.01) |  |
| B*35:22g | 1 (0.01) | 4 (<0.01) |  |
| B*35:439g | 1 (0.01) | 19 (<0.01) |  |
| B*35:80g | 1 (0.01) | 3 (<0.01) |  |
| B*37:01g | 139 (0.98) | 6053 (1.02) |  |
| B*37:07g | 2 (0.01) | 3 (<0.01) |  |
| B*37:10g | 1 (0.01) | 1 (<0.01) |  |
| B*38:01g | 243 (1.7) | 11092 (1.86) |  |
| B*38:02g | 2 (0.01) | 114 (0.02) |  |
| B*39:01g | 145 (1.02) | 4604 (0.77) |  |
| B*39:02g | 32 (0.22) | 1144 (0.19) |  |
| B*39:03g | 48 (0.34) | 1591 (0.27) |  |
| B*39:05g | 111 (0.78) | 3884 (0.65) |  |
| B*39:06g | 69 (0.48) | 3230 (0.54) |  |
| B*39:09g | 60 (0.42) | 2128 (0.36) |  |
| B*39:10g | 37 (0.26) | 1259 (0.21) |  |
| B*39:12g | 9 (0.06) | 241 (0.04) |  |
| B*39:13g | 66 (0.46) | 2260 (0.38) |  |
| B*39:14g | 5 (0.04) | 107 (0.02) |  |
| B*39:24g | 8 (0.06) | 315 (0.05) |  |
| B*39:37g | 2 (0.01) | 142 (0.02) |  |
| B*40:01g | 251 (1.76) | 10289 (1.73) |  |
| B*40:02g | 297 (2.08) | 11270 (1.89) |  |
| B*40:03g | 13 (0.09) | 167 (0.03) |  |
| B*40:04g | 140 (0.98) | 5383 (0.9) |  |
| B*40:06g | 27 (0.19) | 976 (0.16) |  |
| B*40:08g | 3 (0.02) | 7 (<0.01) |  |
| B*40:11g | 2 (0.01) | 162 (0.03) |  |
| B*40:129g | 4 (0.03) | 203 (0.03) |  |
| B*40:12g | 2 (0.01) | 22 (<0.01) |  |
| B*40:16g | 12 (0.08) | 265 (0.04) |  |
| B*40:334g | 1 (0.01) | 0 (<0.01) |  |
| B*41:01g | 106 (0.74) | 4889 (0.82) |  |
| B*41:02g | 69 (0.48) | 2797 (0.47) |  |
| B*42:01g | 175 (1.23) | 8750 (1.47) |  |
| B*42:02g | 56 (0.39) | 1858 (0.31) |  |
| B*44:02g | 537 (3.77) | 23870 (4) |  |
| B*44:03g | 788 (5.53) | 37043 (6.21) |  |
| B*44:04g | 4 (0.03) | 254 (0.04) |  |
| B*44:05g | 28 (0.2) | 1442 (0.24) |  |
| B*44:07g | 1 (0.01) | 3 (<0.01) |  |
| B*45:01g | 296 (2.08) | 11654 (1.95) |  |
| B*46:01g | 15 (0.11) | 160 (0.03) | <0.0001 |
| B*47:01g | 35 (0.25) | 1246 (0.21) |  |
| B*48:01g | 22 (0.15) | 362 (0.06) | 0.0017 |
| B*48:02g | 112 (0.79) | 3840 (0.64) |  |
| B*48:03g | 10 (0.07) | 418 (0.07) |  |
| B*49:01g | 395 (2.77) | 16482 (2.76) |  |
| B*50:01g | 235 (1.65) | 12333 (2.07) | 0.0386 |
| B*50:02g | 65 (0.46) | 2569 (0.43) |  |
| B*51:01g | 1043 (7.32) | 44861 (7.52) |  |
| B*51:02g | 2 (0.01) | 144 (0.02) |  |
| B*51:04g | 31 (0.22) | 838 (0.14) |  |
| B*51:05g | 1 (0.01) | 58 (0.01) |  |
| B*51:07g | 17 (0.12) | 649 (0.11) |  |
| B*51:08g | 22 (0.15) | 850 (0.14) |  |
| B*51:09g | 2 (0.01) | 52 (0.01) |  |
| B*51:105g | 1 (0.01) | 1 (<0.01) |  |
| B*51:65g | 2 (0.01) | 65 (0.01) |  |
| B*51:75g | 3 (0.02) | 141 (0.02) |  |
| B*52:01g | 312 (2.19) | 11912 (2) |  |
| B*52:02g | 2 (0.01) | 17 (<0.01) |  |
| B*52:112g | 1 (0.01) | 2 (<0.01) |  |
| B*52:21g | 2 (0.01) | 46 (0.01) |  |
| B*53:01g | 432 (3.03) | 16827 (2.82) |  |
| B*54:01g | 21 (0.15) | 210 (0.04) | <0.0001 |
| B*55:01g | 122 (0.86) | 5947 (1) |  |
| B*55:02g | 11 (0.08) | 109 (0.02) |  |
| B*56:01g | 40 (0.28) | 1834 (0.31) |  |
| B*56:03g | 2 (0.01) | 5 (<0.01) |  |
| B*56:15g | 2 (0.01) | 10 (<0.01) |  |
| B*57:01g | 243 (1.7) | 11059 (1.85) |  |
| B*57:02g | 20 (0.14) | 899 (0.15) |  |
| B*57:03g | 111 (0.78) | 4841 (0.81) |  |
| B*57:04g | 2 (0.01) | 71 (0.01) |  |
| B*58:01g | 259 (1.82) | 12350 (2.07) |  |
| B*58:02g | 131 (0.92) | 5683 (0.95) |  |
| B*59:01g | 4 (0.03) | 62 (0.01) |  |
| B*67:01g | 4 (0.03) | 103 (0.02) |  |
| B*73:01g | 16 (0.11) | 428 (0.07) |  |
| B*78:01g | 13 (0.09) | 572 (0.1) |  |
| B*81:01g | 81 (0.57) | 3390 (0.57) |  |
| B*82:01g | 11 (0.08) | 319 (0.05) |  |
| C*01:02g | 360 (2.77) | 13537 (2.32) | 0.023 |
| C*01:03g | 1 (0.01) | 7 (<0.01) |  |
| C*01:22g | 1 (0.01) | 141 (0.02) |  |
| C*02:02g | 838 (6.44) | 36869 (6.32) |  |
| C*02:05g | 1 (0.01) | 3 (<0.01) |  |
| C*02:14g | 3 (0.02) | 105 (0.02) |  |
| C*02:27g | 1 (0.01) | 2 (<0.01) |  |
| C*03:02g | 93 (0.71) | 4124 (0.71) |  |
| C*03:03g | 514 (3.95) | 23406 (4.01) |  |
| C*03:04g | 854 (6.56) | 35315 (6.05) |  |
| C*03:05g | 7 (0.05) | 52 (0.01) |  |
| C*03:07g | 1 (0.01) | 70 (0.01) |  |
| C*03:16g | 4 (0.03) | 90 (0.02) |  |
| C*03:38g | 4 (0.03) | 131 (0.02) |  |
| C*03:41g | 1 (0.01) | 22 (<0.01) |  |
| C*03:44g | 1 (0.01) | 4 (<0.01) |  |
| C*03:47g | 1 (0.01) | 5 (<0.01) |  |
| C*04:01g | 2162 (16.62) | 96604 (16.55) |  |
| C*04:03g | 3 (0.02) | 86 (0.01) |  |
| C*04:04g | 1 (0.01) | 98 (0.02) |  |
| C*04:07g | 14 (0.11) | 375 (0.06) |  |
| C*04:08g | 1 (0.01) | 22 (<0.01) |  |
| C*04:13g | 4 (0.03) | 123 (0.02) |  |
| C*04:29g | 1 (0.01) | 0 (<0.01) |  |
| C*05:01g | 658 (5.06) | 30333 (5.2) |  |
| C*05:08g | 1 (0.01) | 16 (<0.01) |  |
| C*05:09g | 5 (0.04) | 42 (0.01) |  |
| C*06:02g | 993 (7.63) | 46867 (8.03) |  |
| C*07:01g | 1447 (11.12) | 69925 (11.98) |  |
| C*07:02g | 1142 (8.78) | 51463 (8.82) |  |
| C*07:04g | 129 (0.99) | 6089 (1.04) |  |
| C*07:05g | 3 (0.02) | 164 (0.03) |  |
| C*07:07g | 11 (0.08) | 258 (0.04) |  |
| C*07:123g | 1 (0.01) | 5 (<0.01) |  |
| C*07:93g | 4 (0.03) | 10 (<0.01) |  |
| C*08:01g | 25 (0.19) | 896 (0.15) |  |
| C*08:02g | 694 (5.33) | 29273 (5.01) |  |
| C*08:03g | 26 (0.2) | 526 (0.09) | 0.0023 |
| C*08:04g | 40 (0.31) | 1678 (0.29) |  |
| C*08:31g | 1 (0.01) | 3 (<0.01) |  |
| C*12:02g | 122 (0.94) | 4750 (0.81) |  |
| C*12:03g | 597 (4.59) | 27417 (4.7) |  |
| C*12:12g | 5 (0.04) | 180 (0.03) |  |
| C*14:02g | 285 (2.19) | 12416 (2.13) |  |
| C*14:03g | 22 (0.17) | 906 (0.16) |  |
| C*15:02g | 558 (4.29) | 23985 (4.11) |  |
| C*15:03g | 11 (0.08) | 266 (0.05) |  |
| C*15:04g | 7 (0.05) | 301 (0.05) |  |
| C*15:05g | 130 (1) | 5015 (0.86) |  |
| C*15:06g | 2 (0.02) | 62 (0.01) |  |
| C*15:08g | 2 (0.02) | 184 (0.03) |  |
| C*15:10g | 1 (0.01) | 1 (<0.01) |  |
| C*15:31g | 1 (0.01) | 1 (<0.01) |  |
| C*16:01g | 634 (4.87) | 30967 (5.3) |  |
| C*16:02g | 90 (0.69) | 4536 (0.78) |  |
| C*16:04g | 13 (0.1) | 553 (0.09) |  |
| C*17:01g | 344 (2.64) | 16808 (2.88) |  |
| C*18:01g | 134 (1.03) | 5655 (0.97) |  |
| C*18:04g | 1 (0.01) | 15 (<0.01) |  |
| DPA1*01:03g | 7675 (68.17) | 69242 (67.52) |  |
| DPA1*01:04g | 36 (0.32) | 343 (0.33) |  |
| DPA1*01:05g | 9 (0.08) | 90 (0.09) |  |
| DPA1*01:141g | 1 (0.01) | 0 (<0.01) |  |
| DPA1*01:27g | 1 (0.01) | 3 (<0.01) |  |
| DPA1*02:01g | 2360 (20.96) | 22514 (21.95) |  |
| DPA1*02:02g | 793 (7.04) | 7228 (7.05) |  |
| DPA1*02:03g | 2 (0.02) | 39 (0.04) |  |
| DPA1*02:09g | 1 (0.01) | 2 (<0.01) |  |
| DPA1*03:01g | 328 (2.91) | 2583 (2.52) |  |
| DPA1*03:02g | 4 (0.04) | 28 (0.03) |  |
| DPA1*04:01g | 48 (0.43) | 460 (0.45) |  |
| DPB1*01:01g | 1191 (9.62) | 57300 (9.83) |  |
| DPB1*02:01g | 1819 (14.69) | 88498 (15.17) |  |
| DPB1*02:02g | 95 (0.77) | 3988 (0.68) |  |
| DPB1*03:01g | 1007 (8.13) | 46551 (7.98) |  |
| DPB1*04:01g | 3219 (26) | 158838 (27.24) |  |
| DPB1*04:02g | 2078 (16.78) | 93161 (15.97) |  |
| DPB1*05:01g | 302 (2.44) | 12634 (2.17) |  |
| DPB1*06:01g | 103 (0.83) | 5245 (0.9) |  |
| DPB1*08:01g | 1 (0.01) | 3 (<0.01) |  |
| DPB1*09:01g | 71 (0.57) | 2919 (0.5) |  |
| DPB1*10:01g | 262 (2.12) | 13004 (2.23) |  |
| DPB1*11:01g | 291 (2.35) | 13431 (2.3) |  |
| DPB1*1264:01g | 1 (0.01) | 0 (<0.01) |  |
| DPB1*13:01g | 448 (3.62) | 19948 (3.42) |  |
| DPB1*14:01g | 429 (3.46) | 17861 (3.06) |  |
| DPB1*15:01g | 59 (0.48) | 2929 (0.5) |  |
| DPB1*156:01g | 1 (0.01) | 0 (<0.01) |  |
| DPB1*16:01g | 51 (0.41) | 2407 (0.41) |  |
| DPB1*17:01g | 480 (3.88) | 23014 (3.95) |  |
| DPB1*18:01g | 153 (1.24) | 6383 (1.09) |  |
| DPB1*19:01g | 44 (0.36) | 2246 (0.39) |  |
| DPB1*20:01g | 28 (0.23) | 1280 (0.22) |  |
| DPB1*23:01g | 20 (0.16) | 1205 (0.21) |  |
| DPB1*26:01g | 5 (0.04) | 137 (0.02) |  |
| DPB1*27:01g | 90 (0.73) | 3225 (0.55) |  |
| DPB1*271:01g | 1 (0.01) | 7 (<0.01) |  |
| DPB1*29:01g | 21 (0.17) | 664 (0.11) |  |
| DPB1*30:01g | 14 (0.11) | 590 (0.1) |  |
| DPB1*304:01g | 1 (0.01) | 10 (<0.01) |  |
| DPB1*329:01g | 1 (0.01) | 53 (0.01) |  |
| DPB1*34:01g | 10 (0.08) | 744 (0.13) |  |
| DPB1*35:01g | 11 (0.09) | 415 (0.07) |  |
| DPB1*382:01N | 2 (0.02) | 32 (0.01) |  |
| DPB1*39:01g | 16 (0.13) | 804 (0.14) |  |
| DPB1*40:01g | 17 (0.14) | 978 (0.17) |  |
| DPB1*45:01g | 6 (0.05) | 261 (0.04) |  |
| DPB1*46:01g | 3 (0.02) | 31 (0.01) |  |
| DPB1*55:01g | 2 (0.02) | 38 (0.01) |  |
| DPB1*57:01g | 1 (0.01) | 5 (<0.01) |  |
| DPB1*59:01g | 1 (0.01) | 12 (<0.01) |  |
| DPB1*60:01g | 1 (0.01) | 4 (<0.01) |  |
| DPB1*61:01N | 2 (0.02) | 80 (0.01) |  |
| DPB1*63:01g | 1 (0.01) | 165 (0.03) |  |
| DPB1*79:01g | 1 (0.01) | 2 (<0.01) |  |
| DPB1*80:01g | 4 (0.03) | 43 (0.01) |  |
| DPB1*81:01g | 2 (0.02) | 14 (<0.01) |  |
| DPB1*85:01g | 15 (0.12) | 728 (0.12) |  |
| DPB1*99:01g | 1 (0.01) | 52 (0.01) |  |
| DQA1*01:01g | 1943 (15.09) | 28850 (15.22) |  |
| DQA1*01:02g | 2251 (17.48) | 33488 (17.67) |  |
| DQA1*01:03g | 868 (6.74) | 14249 (7.52) | 0.0097 |
| DQA1*01:06g | 1 (0.01) | 7 (<0.01) |  |
| DQA1*01:10g | 14 (0.11) | 202 (0.11) |  |
| DQA1*01:159g | 1 (0.01) | 0 (<0.01) |  |
| DQA1*01:25g | 1 (0.01) | 1 (<0.01) |  |
| DQA1*02:01g | 1445 (11.22) | 23782 (12.55) | <0.0001 |
| DQA1*03:01g | 2098 (16.29) | 28147 (14.85) | <0.0001 |
| DQA1*03:86g | 1 (0.01) | 0 (<0.01) |  |
| DQA1*04:01g | 963 (7.48) | 12220 (6.45) | <0.0001 |
| DQA1*04:06g | 3 (0.02) | 15 (0.01) |  |
| DQA1*05:01g | 3223 (25.03) | 47488 (25.06) |  |
| DQA1*05:02g | 13 (0.1) | 171 (0.09) |  |
| DQA1*06:01g | 52 (0.4) | 853 (0.45) |  |
| DQB1*02:01g | 3041 (19.15) | 119800 (20.53) | 0.0003 |
| DQB1*02:03g | 2 (0.01) | 167 (0.03) |  |
| DQB1*03:01g | 3057 (19.26) | 109172 (18.71) |  |
| DQB1*03:02g | 1599 (10.07) | 57336 (9.83) |  |
| DQB1*03:03g | 509 (3.21) | 18158 (3.11) |  |
| DQB1*03:04g | 50 (0.31) | 1237 (0.21) |  |
| DQB1*03:05g | 8 (0.05) | 277 (0.05) |  |
| DQB1*04:01g | 32 (0.2) | 395 (0.07) | <0.0001 |
| DQB1*04:02g | 1188 (7.48) | 40397 (6.92) |  |
| DQB1*04:47g | 2 (0.01) | 52 (0.01) |  |
| DQB1*05:01g | 2165 (13.64) | 77933 (13.36) |  |
| DQB1*05:02g | 422 (2.66) | 15467 (2.65) |  |
| DQB1*05:03g | 387 (2.44) | 13803 (2.37) |  |
| DQB1*05:04g | 3 (0.02) | 140 (0.02) |  |
| DQB1*05:16g | 1 (0.01) | 2 (<0.01) |  |
| DQB1*05:97g | 1 (0.01) | 2 (<0.01) |  |
| DQB1*06:01g | 155 (0.98) | 4821 (0.83) |  |
| DQB1*06:02g | 1642 (10.34) | 58493 (10.03) |  |
| DQB1*06:03g | 915 (5.76) | 37939 (6.5) | 0.0028 |
| DQB1*06:04g | 432 (2.72) | 17305 (2.97) |  |
| DQB1*06:05g | 1 (0.01) | 0 (<0.01) |  |
| DQB1*06:08g | 8 (0.05) | 178 (0.03) |  |
| DQB1*06:09g | 239 (1.51) | 8413 (1.44) |  |
| DQB1*06:11g | 16 (0.1) | 433 (0.07) |  |
| DQB1*06:20g | 1 (0.01) | 1 (<0.01) |  |
| DRB1*01:01g | 896 (5.34) | 30648 (5.14) |  |
| DRB1*01:02g | 731 (4.36) | 24006 (4.03) |  |
| DRB1*01:03g | 108 (0.64) | 3625 (0.61) |  |
| DRB1*03:01g | 1342 (8) | 49134 (8.24) |  |
| DRB1*03:02g | 251 (1.5) | 8768 (1.47) |  |
| DRB1*03:05g | 2 (0.01) | 2 (<0.01) |  |
| DRB1*03:15g | 7 (0.04) | 163 (0.03) |  |
| DRB1*03:189N | 1 (0.01) | 0 (<0.01) |  |
| DRB1*04:01g | 305 (1.82) | 11254 (1.89) |  |
| DRB1*04:02g | 242 (1.44) | 9417 (1.58) |  |
| DRB1*04:03g | 173 (1.03) | 5941 (1) |  |
| DRB1*04:04g | 462 (2.75) | 14201 (2.38) |  |
| DRB1*04:05g | 383 (2.28) | 12840 (2.15) |  |
| DRB1*04:06g | 48 (0.29) | 1441 (0.24) |  |
| DRB1*04:07g | 130 (0.77) | 4790 (0.8) |  |
| DRB1*04:08g | 63 (0.38) | 2117 (0.36) |  |
| DRB1*04:09g | 6 (0.04) | 360 (0.06) |  |
| DRB1*04:10g | 14 (0.08) | 324 (0.05) |  |
| DRB1*04:11g | 380 (2.26) | 12336 (2.07) |  |
| DRB1*04:17g | 1 (0.01) | 3 (<0.01) |  |
| DRB1*04:330g | 1 (0.01) | 2 (<0.01) |  |
| DRB1*07:01g | 1890 (11.26) | 75712 (12.7) | <0.0001 |
| DRB1*08:01g | 316 (1.88) | 11756 (1.97) |  |
| DRB1*08:02g | 260 (1.55) | 7932 (1.33) |  |
| DRB1*08:03g | 81 (0.48) | 2606 (0.44) |  |
| DRB1*08:04g | 373 (2.22) | 11158 (1.87) | 0.0441 |
| DRB1*08:06g | 21 (0.13) | 586 (0.1) |  |
| DRB1*08:07g | 140 (0.83) | 4880 (0.82) |  |
| DRB1*08:10g | 1 (0.01) | 36 (0.01) |  |
| DRB1*08:18g | 2 (0.01) | 26 (<0.01) |  |
| DRB1*09:01g | 335 (2) | 11184 (1.88) |  |
| DRB1*10:01g | 340 (2.03) | 12491 (2.09) |  |
| DRB1*11:01g | 1147 (6.83) | 38197 (6.41) |  |
| DRB1*11:02g | 279 (1.66) | 9680 (1.62) |  |
| DRB1*11:03g | 111 (0.66) | 3736 (0.63) |  |
| DRB1*11:04g | 471 (2.81) | 15388 (2.58) |  |
| DRB1*11:09g | 2 (0.01) | 24 (<0.01) |  |
| DRB1*11:10g | 3 (0.02) | 120 (0.02) |  |
| DRB1*11:11g | 1 (0.01) | 16 (<0.01) |  |
| DRB1*11:12g | 1 (0.01) | 16 (<0.01) |  |
| DRB1*11:13g | 3 (0.02) | 160 (0.03) |  |
| DRB1*11:14g | 2 (0.01) | 25 (<0.01) |  |
| DRB1*11:18g | 4 (0.02) | 73 (0.01) |  |
| DRB1*11:19g | 3 (0.02) | 95 (0.02) |  |
| DRB1*12:01g | 264 (1.57) | 9501 (1.59) |  |
| DRB1*12:02g | 21 (0.13) | 653 (0.11) |  |
| DRB1*13:01g | 1061 (6.32) | 41632 (6.98) | 0.0426 |
| DRB1*13:02g | 743 (4.43) | 28217 (4.73) |  |
| DRB1*13:03g | 251 (1.5) | 8429 (1.41) |  |
| DRB1*13:04g | 35 (0.21) | 781 (0.13) |  |
| DRB1*13:05g | 22 (0.13) | 1018 (0.17) |  |
| DRB1*13:07g | 1 (0.01) | 0 (<0.01) |  |
| DRB1*13:111g | 1 (0.01) | 4 (<0.01) |  |
| DRB1*13:11g | 1 (0.01) | 12 (<0.01) |  |
| DRB1*13:16g | 4 (0.02) | 69 (0.01) |  |
| DRB1*13:17g | 1 (0.01) | 5 (<0.01) |  |
| DRB1*13:21g | 1 (0.01) | 5 (<0.01) |  |
| DRB1*13:31g | 2 (0.01) | 87 (0.01) |  |
| DRB1*13:40g | 2 (0.01) | 230 (0.04) |  |
| DRB1*13:56g | 4 (0.02) | 81 (0.01) |  |
| DRB1*13:80g | 2 (0.01) | 35 (0.01) |  |
| DRB1*14:01g | 410 (2.44) | 14317 (2.4) |  |
| DRB1*14:02g | 239 (1.42) | 7549 (1.27) |  |
| DRB1*14:03g | 3 (0.02) | 49 (0.01) |  |
| DRB1*14:04g | 20 (0.12) | 635 (0.11) |  |
| DRB1*14:05g | 5 (0.03) | 65 (0.01) |  |
| DRB1*14:06g | 53 (0.32) | 1502 (0.25) |  |
| DRB1*14:13g | 15 (0.09) | 109 (0.02) |  |
| DRB1*14:21g | 3 (0.02) | 69 (0.01) |  |
| DRB1*14:86g | 1 (0.01) | 0 (<0.01) |  |
| DRB1*15:01g | 918 (5.47) | 35167 (5.9) |  |
| DRB1*15:02g | 138 (0.82) | 4595 (0.77) |  |
| DRB1*15:03g | 602 (3.59) | 19841 (3.33) |  |
| DRB1*15:06g | 1 (0.01) | 61 (0.01) |  |
| DRB1*15:11g | 2 (0.01) | 16 (<0.01) |  |
| DRB1*16:01g | 249 (1.48) | 9027 (1.51) |  |
| DRB1*16:02g | 374 (2.23) | 13197 (2.21) |  |
| DRB1*16:05g | 1 (0.01) | 4 (<0.01) |  |
